# The mediating effect of health behaviors on the association between job strain and mental health outcome: a national survey of police officers

**DOI:** 10.1038/s41598-024-60746-8

**Published:** 2024-05-01

**Authors:** Ping-Yi Lin, Pochang Tseng, Wen-Miin Liang, Wen-Yu Lin, Yen-Po Cheng, Hsien-Wen Kuo

**Affiliations:** 1 Department of Nursing, Hungkuang University, Taichung, Taiwan; 2 Department of Medical Research, China Medical University Hospital, Taichung, Taiwan; 3 Institute of Environmental and Occupational Health Sciences, National Yang Ming Chao Tung University, No.155, Sec.2, Linong Street, Taipei, 112 Taiwan (ROC); 4 Health Promotion Administration, Ministry of Health and Welfare, Taipei, Taiwan; 5 Department of Health Services Administration, China Medical University, Taichung, Taiwan; 6 Division of Neurosurgery, Department of Surgery, Changhua Christian Medical Foundation Yuanlin Christian Hospital, Changhua, Taiwan; 7 Resource Circulation Administration, Ministry of Environment, Taipei, Taiwan

**Keywords:** Environmental factor, Health behavior, Psychological health, Police officers, Health occupations, Human behaviour

## Abstract

Police officers often face emotionally challenging interpersonal situations and numerous studies have demonstrated that policing is a stressful occupation. A study revealed a significant positive correlation between emotional demands among police officers and emotional dissonance, as well as burnout. Health-promoting behaviors can contribute to better overall health outcomes and reduce the risk of developing health problems, but there is limited research evaluating the association of job strain and health behaviors with mental health outcomes in police officers. The objective of this study was to assess the job strain associated with mental health mediated by health behaviors in Taiwanese police officers. This was a cross-sectional quantitative study conducted in Oct 2016. A total of 41,871 police officers (response rate was 79.7%) participated questionnaire that consisted of demographic information, job characteristics, health behaviors, and mental component summary (MCS) scores of the Short-Form Health Survey. Independent t-tests and one-way analysis of variance (One-way ANOVA) were conducted to assess the differences in mean MCS scores across various demographics, health behavior, and job characteristics. Multivariate regression analyses were used to assess the relationship between job strain and health behaviors with mental health outcomes. MCS scores were associated with job characteristics and health behaviors among police officers except for gender. After adjusting for covariates, multivariate analysis indicated that police officers with high job demands and high job strain index exhibited poor MCS scores. Job strain was significantly associated with MCS mediated by health behaviors (consumption of fruits and vegetables, and physical activity) in Taiwanese police officers. Since regular physical activity and increased vegetable and fruit consumption might alleviate the effects of job strain on mental health status, it is recommended that institutional policies be established to promote health-enhancing behaviors among police officers.

## Introduction

While high job demands and low job control are recognized stressors across various professions, the nature of law enforcement introduces unique stressors specific to policing. Police officers often face emotionally challenging interpersonal situations, such as encounters involving violence and interactions with victims of crime or accidents daily at work. Numerous studies^1^ have demonstrated that policing is a stressful occupation. Police officers’ exposure to work injuries was appraised as the largest stressor, while job pressure was reported as the most frequent stressor. Consequently, police officers often suffer a variety of physiological, psychological, and behavioral effects and symptoms. A study revealed a significant positive correlation between emotional demands among police officers and emotional dissonance, as well as burnout^2^. Schaible and Gecas reported emotional work demands and value dissonance heightened depersonalizing burnout among police officers^3^.

It has been reported that job stress and lack of support were associated with poor physical and mental health in Norwegian police officers^4^. Regarding mental health problems, the prevalence of depression in police officers was 22.8% in Sri Lanka^5^ and 21.6% in Taiwan^6^. Besides, high job demands, poor interpersonal relationships, low job control, and poor adaptation to change were related to poor mental health status among UK prison officers^7^. In English police officers, 27% of them reported long working hours (≥ 49 h/week) and had significantly high rates of psychological distress^8^. Work environment factors seem to be associated with a variety of psychological distress in police officers.

In terms of job characteristics, the demand/control model has been widely used to test work-related stress. High job demands and low job control (autonomy) have been reported to be associated with increased risks of obesity in male Japanese workers^9^ and coronary heart disease (CHD) among British civil servants^10^. Employees with high-strain jobs (low control/high demands) have been suggested to have increased risks of leisure-time physical inactivity^11,12^ and be associated with higher BMI in Finnish public sector employees^13^.

Police, often confronted with highly stressful situations and burnout risk, are instrumental in saving lives in some instances but can also make individuals involved feel as though they are being treated like criminals^14,15^. The DRIVE model was applied to gauge work-related stress among Jamaica police officers, assessing the direct effects of work conditions on well-being. Additionally, it explored the mediating role of perceived job stress and job satisfaction in linking work conditions to well-being^16^. Employees with a high job strain tend to have unhealthy lifestyles. Japanese police officers with a high job strain had an increased risk of CHD through work-related mediating factors, such as long working hours and unhealthy behaviors (drinking and physical inactivity)^17^. Conversely, a positive relationship between psychological well-being and lifestyle factors has been reported, and promoting health behaviors, including increasing physical activity, the consumption of fruits and vegetables (CFV), and controlling smoking, may provide an opportunity for increasing psychological well-being^18^. Police officers experiencing poor mental health were found to have a higher likelihood of engaging in multiple health risk behaviors, including alcohol use, poor dietary habits, smoking, and low physical activity, compared to their mentally healthy counterparts. Additionally, individuals reporting high strain were more inclined to exhibit low physical activity and maintain a poor diet compared to those reporting low strain^19^. It is putative that employees with a high job strain may be at an increased risk of poor health outcomes directly or through mediating factors, such as health behaviors. However, limited evidence has shown the mediation effects of health behaviors to assess the association between job stress and mental health outcomes in police officers. In addition, there was no previous research to assess the direct and indirect effects of job strain and health behaviors on mental health outcomes using the structural equation model (SEM). Therefore, the results are presented in two parts. In the first part, there are self-reported job characteristics and mental health status among police officers. The second part of the study assesses the direct and indirect effects of job strain and mental health outcomes mediated by health behaviors using SEM.

## Methods

### Study population

This was a cross-sectional quantitative study conducted in Oct 2016. The data was collected using a self-administered questionnaire that dealt with demographic information, health behavior, job characteristics, and general health well-being. The study had ethical approval from the China Medical University (CMUH105-REC3-091). All methods were performed in accordance with the Declaration of Helsinki for research involving human subjects. Registered police officers were invited to participate in the study by completing an anonymously distributed questionnaire via mail. Participation was voluntary and signed informed consent was obtained. Eligible participants were those employed as police officers at the time of the study. A total of 53,203 questionnaires were mailed to 89 institutions, encompassing 48,946 for males and 4257 for females. The overall response rate was 79.7% (42,402 out of 53,203). After excluding invalid responses, the final response rate stood at 78.7% (41,871 out of 53,203). Consequently, 41,871 police officers were included in the study’s analysis. The reasons for non-response included vacations, requested time off, and limited time to fill out the questionnaire, and they did not affect the study’s objective. Participant demographic characteristics are presented in Table 1.

**Table 1:** Demographics and job characteristics on mental component summary (MCS) scores among police officers.

	n (%)	Mean (SD)	*p*
Gender			0.1878
Women	6342 (15.2)	59.51 (16.13)	
Men	35,529 (84.8)	59.21 (16.71)	
Age (years)			< 0.001
< 30	7480 (17.9)	57.78 (17.39)	
30–44	17,813 (42.5)	58.47 (16.56)	
≥ 45	16,578 (39.6)	60.77 (16.22)	
Education level			< 0.001
Senior high school	6288 (15.0)	57.80 (17.55)	
Undergraduate	33,273 (79.5)	59.25 (16.48)	
Graduate	2310 (5.5)	63.35 (15.40)	
Marital status			< 0.001
Unmarried	11,133 (26.6)	58.12 (17.14)	
Married	28,766 (68.7)	59.79 (16.36)	
Other	1972 (4.7)	57.85 (17.08)	
Governmental employment			< 0.001
Local	33,612 (80.3)	58.61 (16.74)	
Central	8259 (19.7)	61.88 (15.89)	
Supervision position			< 0.001
No	35,481 (84.7)	58.85 (16.64)	
Yes	6390 (15.3)	61.50 (16.35)	
Nightshift			< 0.001
No	22,241 (53.1)	60.40 (16.58)	
Yes	19,630 (46.9)	57.97 (16.58)	

### Measurement of job strain index (JSI)

Job content was measured using Karasek’s Job Content Questionnaire (JCQ). The occupational JCQ-Taiwan version was obtained from Cheng’s study^20^. This tool comprises of 22 items and three dimensions: job control, job demand, and job support. Job demand is measured by eight items, including time pressure, high complexity, work pressure, exhaustive work, heavy work, limited time and staff to work, and poorly defined tasks. The maximum score was 32, and a higher score represented a higher job demand level. The job control scale is the sum of two subscales including skill discretion and decision authority. JSI was calculated by job demand scores divided by job control scores. Scales of job demand, job control, and JSI were classified into three groups based on the upper tertile (Q3) scores for the high group, the tertile of Q1–Q3 for the moderate group, and < 75% (Q1) for the low group. Participants were asked to indicate their job characteristics using a four-point Likert scale ranging from strongly agree (4 points), agree (3 points), disagree (2 points), and strongly disagree (1 point).

### Measurement of health behaviors

Health behaviors only included frequency of physical activity (never, light exercise once or more a week, moderate or higher exercise once or twice a week, and moderate or higher exercise three times or more a week), and CFV in servings per day were reported by the participants. Alcohol consumption (never, sometimes, and almost every day) and cigarette smoking (non-smoker and current smoker) were not included into a discussion of health behaviors.

### Mental health status

We used the Taiwanese version of the 36-item Short-Form Health Survey (SF-36)^21,22^ to assess police quality of life and mental health for the officers in Taiwan. The SF-36 is a measure of overall health status including eight dimensions of health, namely, physical functioning (PF), role limitations due to physical problems (RP), bodily pain (BP), general health (GH), vitality (VT), social functioning (SF), role limitations due to emotional problems (RE), and mental health (MH). These eight scales can be aggregated into two component summary scores, the physical (PCS) and mental (MCS) component summary scores. Higher scores represent better health status. This study only focuses on MCS.

### Statistical analysis

Data is expressed as mean ± standard deviation (SD) for continuous variables. The independent t-test was performed to compare means of MCS scores between two subgroups relating to gender, governmental employment, the position of superior, nightshift, and health behaviors. The one-way analysis of variance (One-way ANOVA) was conducted to compare MCS scores in different subgroups relating to age, education level, marital status, and job stress. Multivariate regression analyses were used to assess the association between work-related health factors and health behaviors with mental health outcomes. All analyses were adjusted for potential confounders, including age, gender, education level, work duration of employment, the position of superior, and marital status. SEM (structural equation model) was used to assess the association between job strain, health behaviors, and mental health outcomes. P values less than 0.05 were considered to be statistically significant. All statistical analyses were performed with the statistical package SAS V.9.4 (SAS Institute, Cary, North Carolina, USA).

### Institutional review board statement

The study had ethical approval from the China Medical University (CMUH105-REC3-091). Participants were also informed that the data would be handled confidentially.

## Results

Table 1 shows demographic and job characteristics on mental component summary (MCS) scores among police officers. Among police officers, the highest level of education was most commonly a bachelor’s degree (79.5%), over 80% of age ≥ 30 years old, and 68.7% with married. In terms of job types and location, the majority of police officers surveyed fell into the following demographics: they worked for local governments (80.3%), were not in managerial positions (84.7%), and worked night shifts (53.1%). Except for gender, all variables have significance in MCS levels. Police officers with better MCS levels were related to ages of 45 and older (60.77 points), graduate education level (63.35 points), married (59.79 points), working for the central government (61.88 points), supervisorial position (61.50 points).

Health behaviors and job-related stress were significantly associated with mental component summary (MCS) levels among police officers in Table 2. In terms of health behaviors, most of them were non-smokers (73.6%), drank a little (87.8%), did not do physical activity at least three times a week (57.3%), and did not consume vegetables and fruits (62.89%). MCS levels among police officers were low among current smokers (57.45 points), drinking more than three days per week (56.29 points), high job demands (53.62 points), and high JSI (55.6 points). In contrast, police officers with better MCS levels were associated with physical activity more than three days per week (62.39 points) and consumption of vegetables and fruits (63.27 points).

**Table 2:** Health behaviors and job stress associated with mental component summary (MCS) scores among police officers.

	n (%)	Mean (SD)	*p*
Smoking			< 0.001
No	30,825 (73.6)	59.91 (16.56)	
Yes	11,046 (26.4)	57.45 (16.68)	
Drinking > 3 days per week			< 0.001
No	36,322 (86.7)	59.71 (16.55)	
Yes	5549 (13.3)	56.29 (16.83)	
Physical activity > 3 days week			< 0.001
No	24,002 (57.3)	56.92 (17.03)	
Yes	17,869 (42.7)	62.39 (15.53)	
Vegetable and fruit consumption			< 0.001
No	26,331 (62.9)	56.89 (17.02)	
Yes	15,540 (37.1)	63.27 (15.12)	
Job demand			< 0.001
Low	7414 (17.3)	61.35 (16.45)	
Moderate	24,817 (58.0)	60.29 (15.30)	
High	10,568 (24.7)	53.62 (17.53)	
Job control			< 0.001
Low	11,160 (26.1)	56.58 (17.94)	
Moderate	20,582 (48.1)	60.44 (15.63)	
High	11,057 (25.8)	59.83 (16.48)	
Job Strain Index			< 0.001
Low	11,206 (26.2)	61.3 (16.18)	
Moderate	21,465 (50.2)	60.8 (15.6)	
High	10,129 (23.6)	55.6 (17.4)	

Table 3 shows the relationship between job-related stress and mental health outcomes. In Model 1, worse MCS levels were related to high job demands (β = − 7.02) and high JSI (β = − 5.27) using multivariate regression analysis adjusted for covariates (age, gender, education level, marital status, governmental employment, supervisorial position, nightshift, smoking status, and drinking status). In model 2 adjusted for all covariates in model 1 and physical activity, police officers with high job demand (β = − 0.693) and high JSI (β = − 5.15) had poor mental health. In model 3 after adjusting for all covariates in model 2 and CFV, police officers with high job demand (β = − 0.65) and high JSI (β = − 4.77) had poor mental health scores Compared to the coefficients in model 2 and model 3, police officers who had physical activity and CFV were likely to have significant effects on mental health status. Importantly, job demand and JSI were significantly associated with MCS levels after adjusting for covariates.

**Table 3:** Job-related stress associated with mental component summary (MCS) scores using multivariate regression analyses.

	Model 1		Model 2		Model 3	
	Coefficient (95% CI)	*p*	Coefficient (95% CI)	*p*	Coefficient (95% CI)	*p*
Job demand						
Low	Ref.		Ref.		Ref.	
Medium	− 0.17	0.368	− 0.33	0.073	− 0.14	0.427
High	− 7.02	< 0.001	− 6.93	< 0.001	− 6.50	< 0.001
Job control						
Low	Ref.		Ref.		Ref.	
Medium	3.72	< 0.001	3.43	< 0.001	3.32	< 0.001
High	3.41	< 0.001	3.29	< 0.001	2.98	< 0.001
Job strain index						
Low	Ref.		Ref.		Ref.	
Medium	− 0.59	0.005	− 0.72	0.001	− 0.49	0.017
High	− 5.27	< 0.001	− 5.15	< 0.001	− 4.77	< 0.001

Model 1: adjusted for potential confounders including age, gender, education level, marital status, governmental employment, supervision position, nightshift, smoking, and drinking status.

Model 2: model 1 plus physical activity.

Model 3: model 2 plus the consumption of fruits and vegetables.

**Figure 1:**
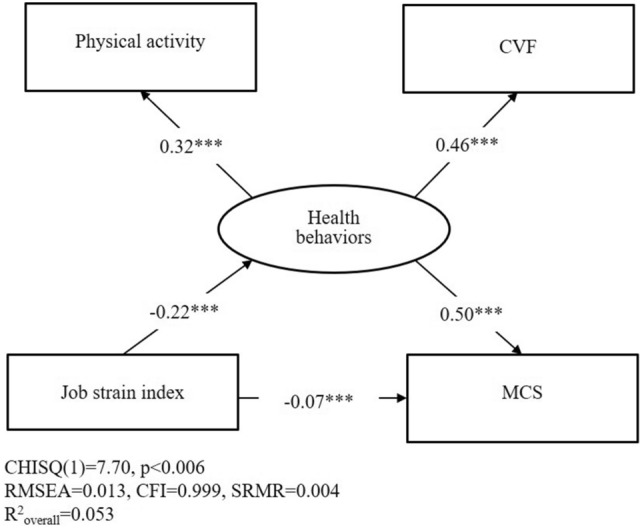
The structural equation model (SEM) was used to assess the direct and indirect effects of job strain index and health behaviors on mental component summary (MCS) scores.

Figure 1 shows the direct and indirect effects of JSI, and health behaviors on MCS scores using SEM. JSI had a negative effect on health behaviors (path coefficient; β = − 0.22, *p* < 0.001), and health behaviors were a positive effect on MCS (β = 0.50, *p* < 0.001). Furthermore, the domain of health behaviors was positively contributing to CFV (β = 0.46, *p* < 0.001) and physical activity (β = 0.32, *p* < 0.001). In addition, the indirect effect of JSI on health behaviors had significant effects on MCS, indicating indirect effect accounted for 61.1%. Health behaviors may play a role in alleviating the effects of JSI on MCS, in particular, the alleviated effect of CFV is higher than those who participate in physical activity.

## Discussion

Evidence shows that exposure to unfavorable psychosocial working conditions such as high demands and low decision latitude are risk factors for mental health and mental disorders^23,24^. Poor psychosocial working conditions and work-related behavioral characteristics, including long working hours, drinking alcohol, and physical inactivity, have been reported to possibly contribute to the higher prevalence of coronary heart diseases among police officers^17^. In the study, we found that worse mental health was related to the location of service, working night shifts, current smoking, drinking more than three days per week, high demands, and high JSI among police officers. Our findings are consistent with previous reports^25^. In terms of health-enhancing behaviors, ages of 45 and older, graduate education level, work for the national government, nightshift, moderate exercise more than three days per week, consumption of vegetables and fruits, and a high decision latitude all tended to be connected to better mental health for police officers. After adjustment for all possible confounders, physical activity and CFV were significantly associated with mental health. The benefits of physical activity for mental health are well known, but the benefits of CFV for mental health are less evaluated. Nutrition plays a crucial role in mental health. Consumption of fruits and vegetables promotes better mental health through various mechanisms, including their nutrient content, antioxidant properties, fiber content, anti-inflammatory effects, and overall nutritional quality^26^. Incorporating a variety of fruits and vegetables into one’s diet can contribute to improved mood and overall mental well-being^27^. A nationally representative panel survey reported that increased fruit and vegetable consumption would increase happiness, life satisfaction, and well-being^28^. Similar results were found in South Asian countries as well^29^.

To the best of our knowledge, this was the first nationwide study of police officers survey to investigate how job demands, job control, and job strain affect mental health status, with a particular focus on the mediating role of health-enhancing behaviors. Although there are some possible mechanisms supportive of a positive relationship between CFV and self-reported mental health status, the pathway is still unclear. Possible mechanisms that may explain the positive link between CFV and mental health include fruits and vegetables containing rich antioxidant components, minerals, and vitamins. These components may help reduce oxidative stress responses^30^. Apart from nutrients, it has been reported that employees who were exposed to high job demands use action planning and coping planning to increase CFV. Coping planning appears to be the most beneficial way to increase CFV^31^. It seems that employees exposed to high job demands and having sufficient intake of fruit and vegetables are associated with better mental health. In addition to mental health, it has been suggested that CFV helps protect against adult depression^32–34^, anxiety^35,36^ and promotes psychological well-being^37^. An extensive cross-sectional and longitudinal analysis based on Australians has shown that increasing CFV may help reduce psychological distress in adults 45 years of age and older^38^. A longitudinal study based on Australian adults has shown that increased CFV is linked to increased happiness, life satisfaction, and subjective well-being^28^. In a longitudinal UK study, a positive correlation between CFV and short-term mental well-being was presented^39^. Within such studies, limited evidence has shown the mediation effect of CFV and physical activity on the relationship between job-related stress and mental health. Whether the health behaviors may mitigate the effect of job strain on mental health, it is believed that people sustainably maintaining health behaviors can enhance their help-seeking and coping mechanism through positive appraisal of their circumstances and reduction in the negative emotional response^40^. The dietary guide recommends consuming at least five servings of fruit and vegetables a day may be beneficial for mental health^41^. Therefore, increasing the frequency of CFV and physical activity can be seen as an inexpensive and effective strategies for promoting mental health at the workplace.

## Conclusion

The present study has several strengths, including a large sample size, a national survey, and a specific job type. This study extends knowledge of health behaviors as a mediator of job strain on mental health outcomes. More research is required into how job-related stress at the workplace threatens physical and mental health and the improvement of health behaviors that might modify this threat, especially encouraging frequency in CFV and physical activity for stressed workers. It can influence the creation of various intervention strategies to improve police officers’ physical and mental health status by future design. However, this study also has some limitations. First, a unique questionnaire was used to assess health behaviors' alleviated effect of job strain on mental health outcomes among police officers. Self-reported data on job strain and health behaviors are generally reliable and recall biases due to individual perception differences are unavoidable. Second, this was a cross-sectional study, so it was impossible to infer causal relationships among the studied variables. MCS could be affected by factors other than health behaviors or job strain, and it has not been measured in this study. Third, the findings of this study are specific to police officers and may not be directly applicable to individuals in other occupations. In addition, the availability of healthy meal options for police officers was not measured, thus, this makes it difficult to understand how the availability of healthy meal options might influence mental health outcomes for police officers. Future well-designed randomized controlled or longitudinal studies are required to investigate the moderating and mediating effects of job strain and health behaviors on police officers' physical and mental health outcomes.

In conclusion, our findings indicate that job strain is significantly associated with self-reported poor mental health status mediated by health behaviors among Taiwanese police officers. Regular physical activity and increased CFV improve mental health and quality of life and may mitigate the effect of job stress on mental health outcomes. Thus, promoting institutional health policy for improving their health-enhancing behavior is suggested for police officers.

## Data Availability

The datasets used and analyzed in the current study are available from the corresponding author on reasonable request.
